# A Doctor’s Name as a Brand: A Nationwide Survey on Registered Clinic Names in Taiwan

**DOI:** 10.3390/ijerph15061134

**Published:** 2018-06-01

**Authors:** Feng-Yuan Chu, Ying-Xiu Dai, Jui-Yao Liu, Tzeng-Ji Chen, Li-Fang Chou, Shinn-Jang Hwang

**Affiliations:** 1 Department of Family Medicine, Taipei Veterans General Hospital, No. 201, Sec. 2, Shi-Pai Road, Taipei 112, Taiwan; steven2259898@gmail.com (F.-Y.C.); ryliu@vghtpe.gov.tw (J.-Y.L.); sjhwang@vghtpe.gov.tw (S.-J.H.); 2 Department of Dermatology, Taipei Veterans General Hospital, No. 201, Sec. 2, Shi-Pai Road, Taipei 112, Taiwan; daiinxiu@gmail.com; 3 School of Medicine, National Yang-Ming University, No. 155, Sec. 2, Linong Street, Taipei 112, Taiwan; 4 Department of Public Finance, National Chengchi University, Taipei 116, Taiwan; lifang@nccu.edu.tw

**Keywords:** names, eponym, private practice, personal branding, Taiwan

## Abstract

In countries where the private clinics of physicians can be freely named, registering a clinic with a physician’s name is one way to make patients familiar with the physician. No previous study had investigated how clinics make use of this method of personal branding. Therefore, the current study analyzed 10,847 private physician Western medicine clinics in Taiwan. Of those clinics, 31.0% (*n* = 3363) were named with a physician’s full name, 8.9% (*n* = 960) with a surname, and 8.1% (*n* = 884) with a given name. The proportion of clinics registered with a physician’s name was lower in rural areas (37.3%) than in urban (48.5%) and suburban areas (49.2%), respectively. Among clinics with only one kind of specialist, a physician’s name was used most frequently in clinics of obstetrics and gynecology (64.9%), otorhinolaryngology (64.1%), and dermatology (63.4%). In Taiwan, fewer than half of clinics used a physician’s name as a brand. The sociocultural or strategic factors and real benefits of doing so could be further studied in the future for a better understanding of healthcare services management.

## 1. Introduction

A good physician-patient relationship is important for diagnosing diseases, dealing with a patient’s worries and expectations, providing appropriate medical treatment, achieving treatment compliance, enhancing patient satisfaction, and ensuring health care quality [1,2,3,4,5,6,7]. The suggested best practice includes a patient becoming well acquainted with his or her physician [8,9], but in reality, most patients can hardly remember their physicians’ names, especially during hospitalizations [10,11]. Such situations might occur less often in physician clinics, where physicians’ names are often included in the registered names of the clinics themselves. Of course, in many countries, e.g., China and Taiwan, the registered name of a clinic may include words other than a physician’s name. As such, the signboards of many clinics in Taiwan do not show any physicians’ names. In the past, two studies have investigated the registered names of psychiatric clinics and hospice wards in Taiwan from the viewpoint of destigmatization [12,13]. No study has yet attempted, however, to investigate the registered names of clinics from a viewpoint of personal branding, a type of branding which views a person himself or herself as a brand with his or her name being a brand name [14,15]. Personal branding could display a distinctive feature [16] and facilitate entry into different markets successfully [15,17]. In the medical environment, personal branding was useful for patients to recognize physicians’ reputation and for physicians to demonstrate self-confidence and enhance professional satisfaction [18]. Different kinds of specialists might have different thoughts about branding. For example, plastic surgeons are frequently mentioned to make use of social media to improve their brands [19] and obstetricians usually care about their reputation and public image [20]. On the other hand, urbanization level as one of major parameters in demography plays an important role in marketing strategy [21]. Our prior studies also showed that urban-rural disparities in availability of clinics and utilization of healthcare did exist in Taiwan [22,23,24,25]. It is interesting to know whether the strategy of personal branding used in clinics differs among different urbanized areas. Therefore, we conducted a nationwide survey of clinic names in Taiwan. The current study aimed to analyze the relationship between personal branding in clinics and probable related factors such as medical specialties and urbanization.

## 2. Materials and Methods

### 2.1. Background

**Figure 1:**
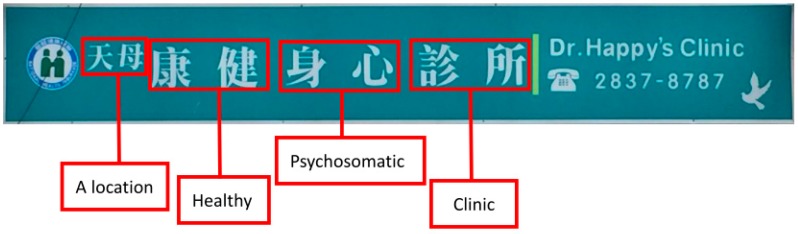
An example of a registered clinic name in Taiwan.

In 2015, a total of 44,192 Western medicine physicians were practicing in Taiwan [26], a country with 23,492,074 inhabitants [27]. While 27,443 of those physicians were working in hospitals, 16,749 were working in private physician clinics with 23 major specialties [26]. The registered names of clinics in Taiwan are regulated by the Enforcement Rules of the Medical Care Act [28]. A physician is free to choose any registered clinic name only if it is not an improper name and no identical name has already existed in the same city or county. In other words, it is not required that a physician’s name be part of a registered clinic name. Figure 1 shows the signboard of a psychiatric clinic in Taiwan as an example. The full name is composed of eight Chinese characters with four phrases (天母 康健 身心 診所). While the fourth phrase means “clinic” and the third means “psychosomatic” (indicating the specialty), the literal meanings of the other two terms (天母 and 康健) are “Tianmu” (a location) and “healthy,” respectively. The physician’s name (黃信得) does not appear in the sign (the name “Dr. Happy” being a fictitious name for marketing purposes).

### 2.2. Data Collection

In August 2015, we obtained the relevant datasets from Taiwan’s Open Government Data platform (https://data.gov.tw/): the basic dataset of healthcare facilities (https://data.gov.tw/node/15393) and the basic dataset of healthcare personnel (https://data.gov.tw/node/15394). The dataset of healthcare facilities contains the identification number, registered name, ownership type, phone number, address, location, specialties, and the counts of various types of healthcare personnel in each facility. The dataset of healthcare personnel contains each healthcare worker’s name, gender, license type, board certifications, registered specialty, and city or county of practice. 

### 2.3. Study Design and Data Extraction

First, we extracted the data of private physician clinics of Western medicine from the dataset of healthcare facilities according to ownership type. We intended to ascertain whether a registered clinic name contained a full name, surname, or given name of a physician. In Chinese, a surname is usually one character and a given name two characters. It was thus easy to recognize a surname or a full name used in a registered name, while difficulties arose with recognizing some given names. Unlike in Western countries, where given names are frequently chosen from among Christian, Biblical, Latin, Romance, Hebrew, Germanic, or Greek names [29], every character of the given names in Chinese-speaking countries is usually chosen on the basis of its pleasant sound (when heard), positive meaning, or beautiful look (when read) [30]. For a clinic name with two characters, therefore, we tried to determine whether the two characters appeared in physicians’ names in the dataset of medical personnel. When the name, specialty, and location of a clinic were all identical to those of a physician, we deemed the clinic in question to be named after the physician.

We then considered two factors that could be associated with the naming of clinics: urbanization and specialty. A clinic’s urbanization level was determined on the basis of the categorization method provided by Liu et al. [31]. In brief, townships in Taiwan were allocated into eight hierarchical levels: highly urbanized towns, medium urbanized towns, emerging towns, general towns and cities, aging towns, agricultural towns, remote towns, and outlying islands. In our study, we further grouped those categories into urban (highly urbanized towns and medium urbanized towns), suburban (emerging towns and general towns/cities), and rural (aging towns, agricultural towns, remote towns, and outlying islands) areas. Furthermore, because a clinic might have several physicians with different specialties, and because a physician might have multiple board certifications, we simplified our analysis of specialty by focusing on those clinics with only one specialty.

### 2.4. Statistical Analysis

The open-source software Perl (version 5.26.1, https://www.perl.org/) was used for data extraction and processing. The statistical analysis was performed using SPSS version 23.0 (IBM, Armonk, NY, USA) and Microsoft Excel 2016 (Microsoft Inc., Redmond, WA, USA).

### 2.5. Ethics Statements

On the basis of the Personal Information Protection Act in Taiwan and the regulations of the institutional review board (IRB) of Taipei Veterans General Hospital (Taipei, Taiwan), the application of publicly accessible data is exempted from the IRB approval process.

## 3. Results

### 3.1. Registered Names at Different Urbanization Levels

Of 368 townships in Taiwan, there were no private physician clinics in only 27 towns. Nearly two thirds of 10,847 private physician clinics were located in urban areas, 28.1% were located in suburban areas, and 6.0% were located in rural areas (Table 1). For 52.0% (*n* = 5640) of clinics, the registered names did not contain any physician’s name, in contrast to the 31.0% (*n* = 3363) that included a physician’s full name, the 8.9% (*n* = 960) that included a physician’s surname, and the 8.1% (*n* = 884) that included a physician’s given name. The proportion of the clinics registered with a physician’s name was lower in rural areas (37.3%) than in urban (48.5%) and suburban areas (49.2%), respectively. The difference was largely attributed to the use of full names, the rate of which was 21.4% among clinics in rural areas and 31.6% among clinics in both urban and suburban areas.

### 3.2. Registered Names by Specialty

**Figure 2:**
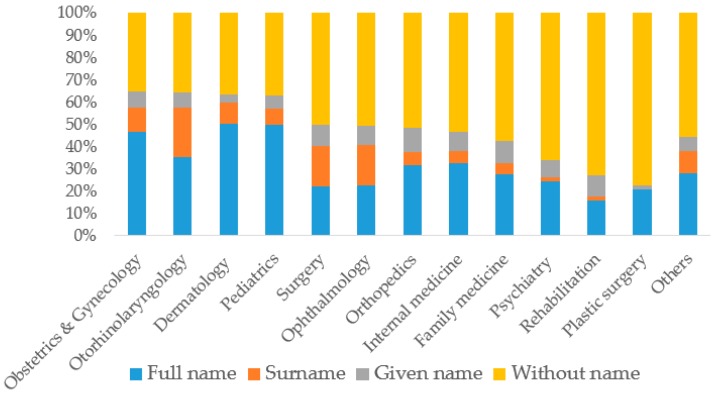
Proportion of the four types of registered names in different one-specialty clinics.

Of the 10,847 clinics, 6596 (60.8%) had only one kind of specialist, 1543 (14.2%) had more than one, and 2708 (24.5%) had none (Table 2). The proportion of registered names including a physician’s name was smaller among the one-specialty clinics (51.9%) than among the multi-specialty clinics (55.2%). Among the one-specialty clinics, a physician’s name was used most frequently in clinics of obstetrics and gynecology (64.9%), followed by otorhinolaryngology (64.1%) and dermatology (63.4%) clinics, in contrast to those of plastic surgery (22.6%), rehabilitation (26.9%), and psychiatry (33.8%) (Figure 2). A specialty was also more commonly utilized in the clinic names of obstetrics and gynecology (89.0%), otorhinolaryngology (94.7%), and dermatology (94.8%) clinics. 

## 4. Discussion

The importance of marketing in healthcare had been neglected until recent decades. It is now recognized, however, that a favorable clinic brand image enhances patient satisfaction and helps to build long-term patient-physician relationships [32]. Naming is a vital element for brand success. In order to create an attractive and potent brand name, some studies have suggested that a name should be unique; positive; contemporary; promotable; impressive; pleasing to the ear; easy to pronounce, remember, and understand; or otherwise suitable to the associated product benefits and corporate image [21,33,34,35]. Our study found that around half of the clinics in Taiwan are named with physicians’ names. Physicians of obstetrics and gynecology, otorhinolaryngology, and dermatology were more likely to use their own names for their clinics. We also found that they all had a higher percentage of utilization of their own specialties in clinic naming. Our findings suggest that physicians’ reputations and specialties are essential elements of clinics’ brands in obstetrics and gynecology, otorhinolaryngology, and dermatology. More research is needed to evaluate the attitudes of physicians in different specialties toward clinic branding. Another finding was that physicians were less likely to register clinics with their names in rural areas. Compared with residents in urban areas, residents in rural areas lack access to health care services. The aforementioned finding thus suggests the hypothesis that physicians’ attitudes toward branding their clinics are associated with the degree of competition in the practice environment. Further studies are needed, however, to confirm this hypothesis. 

We found that lower proportions of clinics of plastic surgery, rehabilitation, and psychiatry included physicians’ names in their names. This finding suggests that in these specialties, factors other than physicians’ reputations might play more important roles in clinic branding. One previous study found, for example, that instead of physicians’ names, words reflecting themes of positive emotional states were frequently used in the names of psychiatry clinics [12]. However, more research should be undertaken to evaluate the patterns of clinics’ names and the underlying mechanisms affecting the naming of clinics with these medical specialties. 

There were some limitations to our study. First, because some clinics were named after ex-physicians’ given names and then owned by other physicians without a change in the registered names, the number of clinics registered with physicians’ names was underestimated in comparison to the number of clinics not registered with physicians’ names. Second, clinics registered with physicians’ nicknames were not analyzed because it was difficult to identify nicknames according to definite rules. Third, although some clinics had no specialties on record, that did not necessarily mean that there was no any specialty in these clinics. A bias in the calculated relationship between clinic naming and specialties might thus exist because of incomplete data. Fourth, some clinics in the dataset were closed and some unrecorded new clinics were opened after 2015. Therefore, the results could not totally represent the current status of clinic naming in Taiwan. Fifth, further studies regarding the actual cause and effect relationships in our results would be helpful because this study was cross-sectional. Last, the real effects of using a physician’s name as a brand are unknown because we lacked for the dataset of incomes and patient volumes from clinics. Further research should be conducted if the dataset is available.

## 5. Conclusions

In Taiwan, fewer than half of clinics used a physician’s name as a brand, especially among rural or one-specialty clinics. The socio-cultural factors, marketing strategies, and the effects of such naming patterns should be further studied in the future for a better understanding of healthcare services management.

## Figures and Tables

**Table 1:** Registered names of private physician clinics at different urbanization levels in Taiwan.

Urbanization Level	No. of Townships	No. of Clinics	Registered Names of Clinics			
			with a Physician’s Full Name	with a Physician’s Surname	with a Physician’s Given Name	Without any Physician’s Name
Urban	69	7142 (100%)	2259 (31.6%)	618 (8.7%)	586 (8.2%)	3679 (51.5%)
Suburban	141	3050 (100%)	964 (31.6%)	294 (9.6%)	242 (7.9%)	1550 (50.8%)
Rural	131	655 (100%)	140 (21.4%)	48 (7.3%)	56 (8.5%)	411 (62.7%)
Total	341	10,847 (100%)	3363 (31.0%)	960 (8.9%)	884 (8.1%)	5640 (52.0%)

**Table 2:** Registered names in clinics with different specialties in Taiwan.

Clinic Type	No. of Clinics	Registered Names of Clinics			
		With a Physician’s Full Name	With a Physician’s Surname	With a Physician’s Given Name	Without any Physician’s Name
One-Specialty	6596 (100%)	2243 (34.0%)	674 (10.2%)	505 (7.7%)	3174 (48.1%)
Obstetrics & Gynecology	564 (100%)	263 (46.7%)	60 (10.6%)	43 (7.6%)	198 (35.1%)
Otorhinolaryngology	970 (100%)	340 (35.0%)	217 (22.4%)	65 (6.7%)	348 (35.9%)
Dermatology	407 (100%)	205 (50.4%)	38 (9.3%)	15 (3.7%)	149 (36.6%)
Pediatrics	912 (100%)	454 (49.8%)	65 (7.1%)	56 (6.1%)	337 (37.0%)
Surgery	231 (100%)	51 (22.1%)	42 (18.2%)	22 (9.5%)	116 (50.2%)
Ophthalmology	676 (100%)	152 (22.5%)	123 (18.2%)	58 (8.6%)	343 (50.7%)
Orthopedics	155 (100%)	49 (31.6%)	9 (5.8%)	17 (11.0%)	80 (51.6%)
Internal medicine	921 (100%)	297 (32.2%)	53 (5.8%)	80 (8.7%)	491 (53.3%)
Family medicine	989 (100%)	271 (27.4%)	50 (5.1%)	98 (9.9%)	570 (57.6%)
Psychiatry	216 (100%)	52 (24.0%)	4 (1.9%)	17 (7.9%)	143 (66.2%)
Rehabilitation	253 (100%)	40 (15.8%)	4 (1.6%)	24 (9.5%)	185 (73.1%)
Plastic surgery	212 (100%)	44 (20.8%)	0 (0%)	4 (1.9%)	164 (77.4%)
Other	90 (100%)	25 (27.8%)	9 (10.0%)	6 (6.7%)	50 (55.5%)
Multi-specialty	1543 (100%)	467 (30.2%)	228 (14.8%)	157 (10.2%)	691 (44.8%)
No specialty	2708 (100%)	653 (24.1%)	58 (2.1%)	222 (8.2%)	1775 (65.6%)
